# Apparent prevalence and selected risk factors of methicillin-resistant *Staphylococcus aureus* and non-*aureus* staphylococci and mammaliicocci in bulk tank milk of dairy herds in Indiana, Ohio, and Michigan

**DOI:** 10.3168/jdsc.2023-0386

**Published:** 2023-10-03

**Authors:** Juliano L. Goncalves, Rinosh Mani, Srinand Sreevatsan, Pamela L. Ruegg

**Affiliations:** 1 Department of Large Animal Clinical Sciences, College of Veterinary Medicine, Michigan State University, East Lansing, MI 48824; 2 Veterinary Diagnostic Laboratory, College of Veterinary Medicine, Michigan State University, Lansing, MI 48910; 3 Department of Pathobiology and Diagnostic Investigation, College of Veterinary Medicine, Michigan State University, East Lansing, MI 48824

## Abstract

The purpose of this study was to determine the apparent prevalence and risk factors of methicillin-resistant *Staphylococcus aureus* and non-*aureus* staphylococci and mammaliicocci (NASM) in bulk tank milk (BTM) obtained from 300 dairy farms that belong to a cooperative collecting milk from Indiana, Michigan, and Ohio. Dairy field personnel recorded information about selected farm level risk factors and collected and froze BTM samples (n = 300) that were sent to Michigan State University researchers. Milk samples were thawed at room temperature and pre-enriched by adding 1 to 4 mL of Mueller-Hinton broth supplemented with 6.5% NaCl and incubated at 37°C for 24 h. Subsequently, 10 µL was plated on mannitol salt agar and Mueller-Hinton agar supplemented with 2.5% NaCl containing 2 mg/L oxacillin and 20 mg/L aztreonam. Colonies that grew on the selective media were subcultured on blood agar and identified using MALDI-TOF mass spectrometry. Phenotypic methicillin resistance was tested using cefoxitin disk diffusion. Conventional PCR was used to detect *mecA* and *mecC* in phenotypically resistant isolates. Of 550 isolates that were obtained from mannitol salt agar plates and 10 isolates from Mueller-Hinton agar plates, 16 species of NASM accounted for 84% of staphylococci, while *S. aureus* accounted for the remaining 16%. Among *S. aureus*, 4 isolates from 4 farms (1.3%) demonstrated phenotypic resistance to methicillin resistance but none carried *mecA* or *mecC* genes. Among NASM, 45 isolates from 40 farms (13.3%) demonstrated phenotypic resistance to methicillin. However, only 13 NASM isolates (7 *Mammaliicoccus sciuri*, 2 *Staphylococcus haemolyticus*, 1 *Mammaliicoccus fleuretti*, 1 *Staphylococcus epidermidis*, 1 *Staphylococcus saprophyticus*, and 1 *Staphylococcus hyicus*) from 13 farms were positive for *mecA*, whereas all were negative for *mecC*. Thus, the prevalence of *mecA*-positive NASM in BTM was 4.3%. Based on molecular results, this study demonstrated a low prevalence of methicillin resistance NASM from BTM samples collected from farms in the Upper Midwest. Dairy farms that contained ≤200 lactating cows and had swine located on the farm had a higher prevalence of methicillin-resistant NASM than smaller farms that did not contain swine.

## Highlights

• No MR *S. aureus* were recovered from BTM of 300 dairy farms and the prevalence of MR NASM was 4.3%
• Our results suggest the use of a pre-enrichment method followed by Mueller-Hinton agar with antibiotics to screen for phenotypic resistance to methicillin, and mannitol salt agar to screen for phenotypic susceptible staphylococci and mammaliicocci.
• Dairy farms that contained ≤200 lactating cows and that had swine located on the farm had a higher prevalence of MR NASM compared with farms that did not contain swine.

## Graphical Abstract

**Figure:**
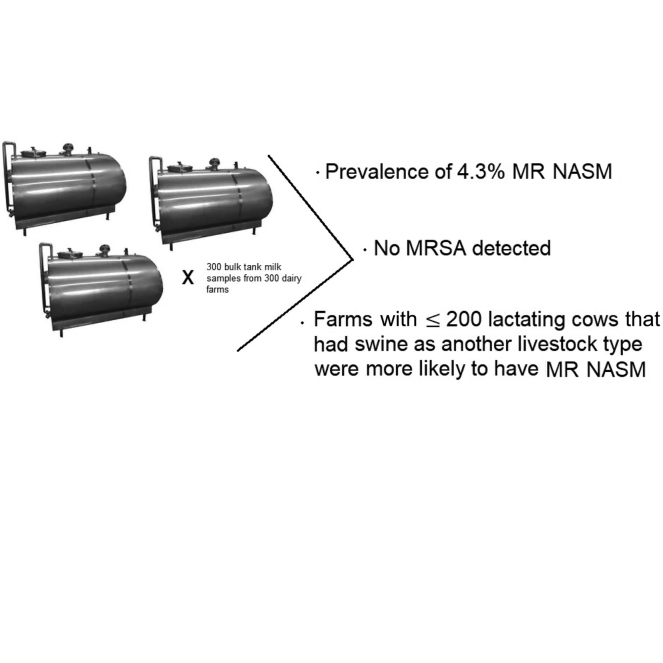
**Summary:** Antimicrobial resistance in agriculture poses potential health risks to animals and humans, making it a serious concern among both consumers and scientists. Non-*aureus* staphylococci and mammaliicocci (NASM) are a highly diverse group of bacteria that are frequently recovered from bulk tank milk (BTM) as well as intramammary infections. This is significant because methicillin-resistant (MR) NASM may serve as a reservoir of resistant genes that pose a risk to humans and animals. The aim of this study was to determine the prevalence of MR *Staphylococcus aureus* and NASM in 300 BTM samples collected from farms across Michigan, Ohio, and Indiana. No MR *S. aureus* were identified; however, 4.3% of BTM contained MR NASM. Dairy farms with ≤200 lactating cows that had swine located on the farm had a higher prevalence of MR NASM than larger farms that did not contain swine.

Antimicrobial resistance poses a risk to both human and animal welfare, and a recently published survey indicated that 91% of consumers viewed antibiotic usage on dairy farms as a threat to their personal health (Wemette et al., 2021). While there is little research indicating that the prevalence of antibiotic-resistant isolates in dairy farms is related to treatment of mastitis (Erskine et al., 2002; Makovec and Ruegg, 2003; Oliver et al., 2011; Oliver and Murinda, 2012), the existence of methicillin resistance in staphylococci in farms is a significant public health concern (Schnitt and Tenhagen, 2020). *Staphylococcus aureus* is an important cause of contagious bovine mastitis, and the prognosis for curing cases caused by β-lactamase–positive strains is poor (Taponen et al., 2003). The poor prognosis of IMI caused by *S. aureus* has resulted in emphasis on prevention of new infections by adoption of best management practices that limit transmission (Ruegg, 2017). In contrast, NAS are minor mastitis pathogens and include >50 species of staphylococci that are widely distributed on teat skin and teat canals (Condas et al., 2017). Recently, 5 common staphylococcal species belonging to the *Staphylococcus sciuri* group (*Staphylococcus sciuri*, *Staphylococcus fleurettii*, *Staphylococcus lentus*, *Staphylococcus stepanovicii*, and *Staphylococcus vitulinus*) were reassigned to the genus *Mammaliicoccus* (Madhaiyan et al., 2020). Non-*aureus* staphylococci and mammaliicocci (**NASM**) are frequently recovered from subclinical mastitis or mild cases of clinical mastitis, often occurring in early lactation (Condas et al., 2017). Fortunately, 44 to 66% of IMI caused by these microorganisms result in spontaneous bacteriological clearance; thus, fewer antibiotics might be used for treatment of NASM (Ruegg, 2018).

Methicillin is a semi-synthetic narrow-spectrum penicillin class drug that was previously used to treat staphylococcal infections in humans, but the development of widespread resistance through expression of penicillin-binding proteins (that might be explained by the rapid degradation in storage), the need for parenteral administration, and the relationship with interstitial nephritis has greatly decreased its usefulness. Although methicillin is not used to treat cattle, development of methicillin resistance is a marker for development of generalized resistance to other β-lactam drugs and methicillin-resistant *S. aureus* (**MRSA**) is considered to be a superbug that threatens human health. Most *S. aureus* occurring on dairy farms are not methicillin-resistant, but transmission of MRSA on dairy farms appears to be bidirectional and there is some evidence that humans can infect cattle with novel strains (Schnitt and Tenhagen, 2020). In a study conducted using 150 bulk tank milk (**BTM**) samples collected from 50 Minnesota dairy farms, researchers reported that methicillin-susceptible *S. aureus* was common (recovered from 84% of BTM), but MRSA were identified in only 4% of samples (Haran et al., 2012).

The prevalence of MRSA varies regionally, and previous studies have demonstrated a very low prevalence of recovery of MRSA from BTM samples collected from US dairy farms (Haran et al., 2012; Cicconi-Hogan et al., 2014). However, NASM can be recovered from most BTM and the prevalence of methicillin-resistant NASM (**MR NASM**) occurring in BTM from dairy herds in the Upper Midwestern region that includes Michigan (**MI**), Ohio (**OH**), and northern Indiana (**IN**) is unknown. We hypothesized that while few samples of BTM will contain MRSA, a greater proportion of BTM samples will harbor MR NASM that may serve as a reservoir for transference of resistance genes to other bacteria. The purpose of this study was to determine the apparent prevalence and risk factors of MRSA and MR NASM from 300 BTM samples collected from dairy farms located in MI, OH, and IN.

One BTM sample per farm was collected from 238 farms from MI, 43 from IN, and 19 from OH of approximately 640 farms that are members of a dairy cooperative and used for this cross-sectional prevalence study. Information about selected risk factors and milk samples were collected by trained dairy field representatives during February to June 2022. Milk samples were frozen at −20°C for up to 5 d until transported to the Michigan State University Top Milk laboratory. Identifying information was removed from milk samples before submission to Michigan State University and researchers remained blind to the identity of the farms that contributed samples; thus, review by an institutional review board was not required.

Screening for MRSA and MR NASM was performed using a 2-step enrichment procedure (Haran et al., 2012; Nemeghaire et al., 2014; Schauer et al., 2021). In step 1, frozen BTM samples were thawed at room temperature and pre-enriched by adding 1 mL of milk to 4 mL of Mueller-Hinton broth (BD Difco) supplemented with 6.5% NaCl and incubated at 37°C for 24 h with shaking at 200 rpm (Fisher and Paterson, 2020). In step 2, 10 µL of pre-enriched sample was streaked onto Mueller-Hinton agar (**MHA**; Neogen) with 2.5% NaCl, 2 mg/L oxacillin (Sigma-Aldrich), and 20 mg/L aztreonam (Supelco, Merck) for initial screening for apparent methicillin-resistant phenotypes (Schauer et al., 2021). After pre-enrichment, a 0.5 McFarland suspension was prepared using sterile saline. Those suspensions were diluted using a 1:10 dilution with saline and 10 µL was streaked onto mannitol salt agar (**MSA**) for initial phenotypic identification of methicillin-resistant staphylococci and mammaliicocci. All colonies that grew on selective media were subcultured on Columbia blood agar and identified using MALDI-TOF MS, Microflex Biotyper (Bruker Daltonics, Billerica, MA) with scores ≥2 indicating a species-level identification (Barcelos et al., 2019). Only one isolate of each species of staphylococci or mammaliicocci was used per enrolled farm. All isolates identified as staphylococci were frozen in brain heart infusion broth (BD Difco) with 20% glycerol at −80°C.

Apparent phenotypic resistance to methicillin was tested using disk diffusion for cefoxitin (30 µg; Oxoid) for *S. aureus*, *Staphylococcus lugdunensis*, and NASM as well as oxacillin (1 µg; Oxoid) for *Staphylococcus pseudintermedius* and *Staphylococcus schleiferi* (CLSI, 2018a,b). In brief, the colony suspension method (CLSI, 2018a) was used to reach 0.5 McFarland standard and the inoculum was spread on MHA supplemented with 2.0% NaCl, cefoxitin disks were applied, and plates were incubated at 35°C for 16 to 24 h (depending on species). Quality control standards (ATCC 51625, 33591, 25923, 29213, and BAA-2313) and breakpoints were used as defined by CLSI (2018b).

For extracting bacterial DNA, frozen bacteria were thawed at room temperature, cultured in brain heart infusion broth (BD Difco), and incubated at 37°C for 16 h. A 1-mL aliquot was centrifuged at 14,000 × *g* for 10 min at 22°C following 2 washing steps of 200 µL of TE buffer (Invitrogen, ThermoFisher Scientific) and centrifugation at 13,000 × *g* for 5 min at 22°C. The pellet was resuspended and homogenized in 50 µL of TE buffer, direct boiled at 95°C for 15 min in heat block (Isotemp, Fisherbrand), and following immediately frozen for 30 min. After this sequence, defrosted samples were centrifuged at 13,000 × *g* for 5 min at 22°C and the supernatant containing the DNA was saved at −20°C. The quality and quantity of the DNA samples was measured using a NanoDrop (ThermoFisher Scientific).

All PCR reactions were adjusted to a volume of 25 µL, which included 5 µL of DNA aliquot (10 µ*M*) as well as 20 µL of a PCR mixture (6.5 µL of DNase-free water, 12.5 µL of Hot Start Taq 2X Master Mix (NewEngland Biolabs), and 0.5 µL each of the forward and reverse primers at concentration of 0.2 µ*M*). All isolates were submitted to PCR amplification for *mecA* detection as described by Gómez-Sanz et al. (2010) with the primer pair forward: GGGATCATAGCGTCATTATTC and reverse: AACGATTGTGACACGATAGCC, and *mecC* detection as described by Cuny et al. (2011) with the primer pair forward: GCTCCTAATGCTAATGCA and reverse: TAAGCAATAATGACTACC. The reactions were performed in a thermal cycler (MiniAmp, ThermoFisher Scientific) under the following conditions: an initial cycle of activation of 94°C for 2 min; 35 cycles of denaturation (94°C for 15 s), annealing temperature (57°C for *mecA* and 51°C for *mecC*, both for 15 s), rapid extension (68°C for 20 s), and one final extension cycle of 72°C for 5 min. *Staphylococcus aureus* strains ATCC 33591 and ATCC BAA-2313 were used as positive controls for *mecA* and *mecC*, respectively. The resulting PCR products were analyzed via electrophoresis on E-Gel EX Double Comb Agarose Gels, 1% (Thermo Fisher Scientific) according to the manufacturer's instructions. Finally, the band size of 527 bp was used to consider *mecA* being detected, whereas 304 bp was used to detect the presence of *mecC* gene. All PCR products that had absence of the genes (no band detected) were double checked in a second round using the standard 1% agarose gel protocol.

Descriptive statistics were performed using SAS version 9.4 (SAS Institute Inc., Cary, NC); strains that had apparent phenotypic resistance tested using disk diffusion and carried the *mecA* or *mecC* genes were considered as true MR NASM as suggested by Schnitt et al. (2021). Apparent prevalence of staphylococci and mammaliicocci species, MRSA, and MR NASM were calculated as a percentage of total farms at risk. Location (MI, IN, or OH), breed (only Holsteins, or others), farm size (small ≤200, or >200, medium 201 to 500, or large >500 lactating cows), and types of livestock (only dairy cows vs. dairy and swine) were used to describe risk factors potentially associated with MRSA and MR NASM using the Fisher's exact test.

In all states, the majority of farms milked primarily Holsteins (Table 1) with ≤200 lactating cows, but larger herds were enrolled in both MI and IN (Table 1). Among all herds, 67% (202/300) contained only dairy animals, 28% (84/300) also had poultry, horses, small ruminants, or beef cattle, and 5% (14/300) housed swine on the premises (Table 1). Among farms that housed swine, 71% milked ≤200 cows.

**Table 1:** Descriptive information of 300 farms belonging to a milk cooperative by herd size, breed, and livestock type

Location	Herd size		Breed		Livestock type		
	Lactating cows	*n*	Holstein	Others	Only dairy	Dairy and others^1^	Dairy and swine^2^
Michigan (n = 238)	≤200	101	97	4	65	28	8
	201–500	89	87	2	75	12	2
	>500	48	48	0	39	7	2
Indiana (n = 43)	≤200	43	36	7	11	31	1
	201–500	0	0	0	0	0	0
	>500	0	0	0	0	0	0
Ohio (n = 19)	≤200	11	10	1	5	5	1
	201–500	4	3	1	3	1	0
	>500	4	4	0	4	0	0

1 Dairy and others including poultry, horses, small ruminants, and beef cattle.

2 Dairy farms that had swine as another livestock type.

Of BTM samples, staphylococci or mammaliicocci species were recovered from 10 of 300 that were streaked on MHA containing antibiotics. Other genera that grew on that media are shown in Table 2. These organisms are expected to grow on MHA plates supplemented with antibiotics as they have been reported to be intrinsically resistant to oxacillin (Fisher and Paterson, 2020) and have had a *mec*-like element identified (Schwendener et al., 2017).

**Table 2:** Staphylococci, mammaliicocci, and other non-staphylococci or mammaliicocci bacteria submitted to a 2-step enrichment procedure, MALDI-TOF MS for species-level identification and antimicrobial susceptibility and PCR testing

Groups of bacteria^1^	MHA^2^		MSA^3^		Antimicrobial susceptibility^4^		Detection of *mecA* gene^5^	
	n	%^6^	n	%	n	%	n	%
Staphylococci and mammaliicocci								
*Staphylococcus aureus*	0		88		4		0	
NASM	10		462		45		13	
*Staphylococcus chromogenes*	0	0.0	202	67.3	1	0.3	0	0.0
*Mammaliicoccus sciuri*	5	1.7	72	24.0	29	9.7	7	2.3
*Staphylococcus simulans*	0	0.0	60	20.0	2	0.7	0	0.0
*Staphylococcus hyicus*	1	0.3	60	20.0	0	0.0	1	0.3
*Staphylococcus microti*	0	0.0	16	5.3	5	1.7	0	0.0
*Staphylococcus xylosus*	0	0.0	14	4.7	0	0.0	0	0.0
*Staphylococcus gallinarum*	0	0.0	10	3.3	2	0.7	0	0.0
*Staphylococcus epidermidis*	0	0.0	8	2.7	1	0.3	1	0.3
*Staphylococcus haemolyticus*	2	0.7	6	2.0	3	1.0	2	0.7
*Staphylococcus borealis*	0	0.0	5	1.7	0	0.0	0	0.0
*Staphylococcus succinus*	0	0.0	4	1.3	0	0.0	0	0.0
*Mammaliicoccus lentus*	0	0.0	3	1.0	0	0.0	0	0.0
*Staphylococcus vitulinus*	0	0.0	1	0.3	0	0.0	0	0.0
*Staphylococcus cohnii*	0	0.0	1	0.3	0	0.0	0	0.0
*Mammaliicoccus fleurettii*	1	0.3	0	0.0	1	0.3	1	0.3
*Staphylococcus saprophyticus*	1	0.3	0	0.0	1	0.3	1	0.3
Subtotal	10		550		49		13	4.3
Non-staphylococci or mammaliicocci bacteria^7^								
*Enterococcus* spp.	203	67.7	2	9.1	—	—	—	—
*Aerococcus* spp.	75	25.0	15	68.2	—	—	—	—
*Macrococcus* spp.	59	19.7	5	22.7	—	—	—	—
*Bacillus* spp.	3	1.0	0	0.0	—	—	—	—
*Candida* spp.	3	1.0	0	0.0	—	—	—	—
*Diutina* spp.	2	0.7	0	0.0	—	—	—	—
*Lactobacillus* spp.	1	0.3	0	0.0	—	—	—	—
*Corynebacterium* spp.	1	0.3	0	0.0	—	—	—	—

1 Isolates submitted to a 2-step enrichment procedure with the inoculation of milk into broth with salt followed by specific agar media and antibiotic or chromogenic agar plate. Isolates were identified at the species level by MALDI-TOF MS.

2 Mueller-Hinton agar (MHA) with 2.5% NaCl, 2 mg/L oxacillin, and 20 mg/L aztreonam.

3 MSA = mannitol salt agar.

4 Apparent phenotypic methicillin resistance was tested using disk diffusion for the surrogate antimicrobial cefoxitin (30 μg) for *S. aureus* and non-*aureus* staphylococci and mammaliicocci (NASM) (CLSI, 2018a,b).

5 All isolates were considered as true methicillin-resistant NASM since they had apparent phenotypic resistance tested using disk diffusion and had methicillin resistance mediated by the *mecA* gene. All isolates were negative for *mecC*.

6 Percentage calculated with bases of 300 dairy farms.

7 Not considered for antimicrobial susceptibility and PCR testing. Of 300 bulk tank milk samples streaked on MHA, MALDI-TOF MS results were suggestive of 60% *Enterococcus faecalis*, 24.7% *Aerococcus viridans*, 17.7% *Macrococcus caseolyticus*, and 7% *Enterococcus faecium*. Of 300 bulk tank milk samples streaked on MSA, MALDI-TOF MS results were suggestive of 59.1% *Aerococcus viridans*, 22.7% *Macrococcus caseolyticus*, 9.1% *Aerococcus urinaeequi*, and 9.1% *Enterococcus faecalis.*

From 300 BTM samples streaked on MSA, 550 staphylococci and mammaliicocci were recovered and one BTM sample did not produce any bacterial growth (Table 2). These results were expected since MSA is selective for any bacteria that tolerates high salt concentrations, including staphylococci and can be used for isolation of both methicillin-resistant and susceptible strains. Sixteen different NASM species accounting for 84% (n = 462 of 550) of staphylococci and mammaliicocci were isolated, while *S. aureus* accounted for the remaining 16% (n = 88 of 550; Table 2).

A wide distribution of species was recovered from the 560 NASM from both MHA and MSA protocols (Table 2). At farm level, *S. chromogenes* was detected in BTM from 67.3% of the farms (n = 202/300), while *S. saprophyticus* was only detected in BTM from one farm. Four *S. aureus* from 4 farms (1.3% of BTM samples) and 45 NASM from 40 farms (13.3%) demonstrated phenotypic resistance to methicillin. Of 550 staphylococci and mammaliicocci obtained from MSA plates, 38 demonstrated phenotypic resistance to methicillin but only 3 were positive for *mecA* and none carried *mecC*. Of 10 staphylococci and mammaliicocci obtained from MHA plates, 3 isolates were susceptible to cefoxitin and 7 demonstrated phenotypic resistance to methicillin. All of those isolates were positive for *mecA* but none carried *mecC*. None of the 4 phenotypically resistant *S. aureus* carried *mecA* or *mecC*. The majority of phenotypically resistant NASM were *M. sciuri* (Table 2). All 13 NASM from 13 farms had *mecA* detected, 10 were isolated from MHA plates (5 *M. sciuri*, 2 *S. haemolyticus*, 1 *M. fleuretti*, 1 *S. saprophyticus*, and 1 *S. hyicus*), while 3 (2 *M. sciuri* and 1 *S. epidermidis*) were isolated from MSA plates (Table 2).

Interestingly, *mecA* was confirmed in 100% of NASM isolated from MHA plates containing antibiotics while *mecA* was confirmed in only 0.65% of NASM isolated (n = 3/462) from MSA (Table 2). Mueller-Hinton agar is considered the standard plating method for detecting resistance isolates. Even though MSA is selective for any bacteria that tolerates high salt concentrations, we were able to isolate approximately 23% of all *mecA-*positive strains using this protocol. We did not compare plating methods because it was not the objective of this study. Based on our results, MSA plates can be used in addition to MHA plates supplemented with antibiotics for screening for methicillin-resistant staphylococci. However, we recognize the potential for missing MR NASM isolates using the MSA plate protocol because one plate may contain hundreds of staphylococcal colonies.

Of 13 MR NASM isolates, 3 (2 *M. sciuri* and 1 *S. hyicus*) were cefoxitin susceptible despite carrying *mecA*. Others have also reported that *M. sciuri* can harbor *mecA* while displaying no phenotypic resistance (Sampimon et al., 2011). This discrepancy between phenotypic and genotypic results has been previously reported for MR NASM by Mahato et al. (2017) who concluded that both susceptibility and genetic tests are needed to properly differentiate methicillin-susceptible and -resistant isolates. To our knowledge, this is the first report of bovine mastitis origin cefoxitin-susceptible MR *Staphylococcus agnetis* (possible *S. hyicus*) obtained from BTM in the United States. Data from the MALDI-TOF MS suggested that the animal origin cefoxitin-susceptible isolate was *S. hyicus*, but researchers have reported that most *S. hyicus* from bovine mastitis cases are actually *S. agnetis* (Adkins et al., 2017). Our results of the sequencing of partial rpoB DNA showed 100% homology to *S. agnetis* while only 96% to *S. hyicus* (data not shown).

*Staphylococcus chromogenes* was the most common NAS recovered from BTM, but it was not the most prevalent MR NASM. Previous researchers have noted that *S. chromogenes* harbors low numbers of resistance genes, whereas MR *S. chromogenes* has been rarely detected in samples from dairy cows (Sampimon et al., 2011; Nobrega et al., 2018). Similar to our results, previous studies that analyzed BTM have reported that *M. sciuri*, *M. fleurettii*, *S. saprophyticus*, *M. lentus*, *S. epidermidis*, and *S. haemolyticus* were the most prevalent MR NASM (Cicconi-Hogan et al., 2014; Fisher and Paterson, 2020; Schauer et al., 2021; Schnitt et al., 2021).

Schauer et al. (2021) also reported that *M. sciuri* was the most prevalent MR mammaliicocci. Methicillin-resistant staphylococci have been documented to transmit SCC*mec* elements to susceptible staphylococcal species among farms, increasing the number of resistance strains (Schnitt et al., 2021). Different SCC*mec* types have been reported for NASM and *S. aureus* isolated from dairy farms and possible transfer of the *mec* gene complex (independent of the SCCmec cassette) was also described in NASM from BTM of dairy farms (Fisher and Paterson, 2020).

As compared with studies of milk from US dairy farms (Haran et al., 2012; Cicconi-Hogan et al., 2014), greater apparent prevalence of MR staphylococci has been reported in BTM from European farms (Fisher and Paterson, 2020; Schauer et al., 2021; Schnitt et al., 2021). Similar to studies in other parts of the world (Fisher and Paterson, 2020) the prevalence of MRSA in BTM from US farms has been very low. Methicillin-resistant *S. aureus* was found in <1% of BTM samples collected from smaller organic and conventional dairy herds (n = 288 farms) located in New York, Wisconsin, and Oregon (Cicconi-Hogan et al., 2014). In a study conducted using 150 BTM samples collected from 50 Minnesota dairy farms, MRSA were identified in only 4% of samples (Haran et al., 2012).

The prevalence of MR NASM has been the focus of few studies worldwide, but among them, 4 studies used BTM samples (Huber et al., 2011; Cicconi-Hogan et al., 2014; Fisher and Paterson, 2020; Schnitt et al., 2021). Direct comparison of results is not possible since data of studies with comparable procedures are lacking. In our study the apparent prevalence of MR NASM in BTM was 4.3% (n = 13/300 farms) but if adjusted similarly according to the sensitivity and specificity of culture-detected MRSA-positive herds as described by Luteijn et al. (2011), the true prevalence values are likely slightly high (i.e., expected to be about 6%, data not shown). The prevalence of MR NASM was similar to reports (4.1%) in the United Kingdom (Fisher and Paterson, 2020) and a previous study performed in the United States (5%; Cicconi-Hogan et al., 2014). Most similar to those dairy farms enrolled in the present study, Cicconi-Hogan et al. (2014) used BTM from 100 organic and conventional farms categorized in 3 herd size (0–99; 100–199; or ≥200 adult cows) from New York, Oregon, and Wisconsin. Fisher and Paterson (2020) used a similar sample size (n = 363) of dairy farms in England and Wales but did not include any explanatory variables. In contrast, 42.1% (8/19) of MR NASM reported in Germany (Schnitt et al., 2021) and 62% (62/100) of MR NASM was reported in Switzerland (Huber et al., 2011). Schnitt et al. (2021) used 19 BTM from 20 different dairy farms of different regions in Germany, but farm level information was only used as a preselection criterion to determine the occurrence of MR NASM in different age groups of cattle (primiparous vs. multiparous or both vs. cows named high risk for having high SCC). Huber et al. (2011) used 100 BTM from 100 different dairy farms in Switzerland but also simultaneously collected swab samples from other livestock types (swine = 241 farms, cows = 253 farms, calves = 187 farms, and chickens = 72 farms). We hypothesize that the greater prevalence of MR NASM found in European studies may be associated a greater proportion of dairy farms containing swine. Huber et al. (2011) found more MR *M. sciuri* and *M. fleurettii* among pigs, cows, calves, and BTM samples while in poultry samples *M. lentus* was more frequently isolated. Although previous researchers did not evaluate livestock as a risk factor, the diversity of species of MR NASM that was most common differed among farms and types of animals, indicating the importance of including livestock as an explanatory variable in future studies.

Differences in prevalence of MRSA and MR NASM reported among studies might be explained by geographic differences, which may be caused by various NASM clones within each country (Cicconi-Hogan et al., 2014; Fisher and Paterson, 2020; Schauer et al., 2021; Schnitt et al., 2021). Within the 3 contiguous states enrolled in our study, we had no difference of MR NASM based on state (IN 0%, n = 0/43, MI 5%, n = 12/238, and OH 5.3%, n = 1/19; *P* = 0.37). Nor did we identify differences based on herd size (only for herds based in MI; small farms, 6.7%, n = 7/101; medium farms, 3.4%, n = 3/89; and large farms, 4.2%, n = 2/48; *P* = 0.55).

Of dairy farms enrolled in our study, 32.7% had some other type of livestock present on their farms (swine, poultry, small ruminants, horses, or beef cattle) but only 5% of dairy farms had pigs as another type of livestock. Based on prior reports associating swine with risk of MRSA (Locatelli et al., 2017; Schnitt and Tenhagen, 2020), we assessed potential associations between MR in BTM and the presence of swine on the dairy farms (n = 14) in comparison to herds that contained only dairy cows or also other livestock (n = 286). Separate analyses were performed for herds that contained ≤200 cows and larger herds (Table 3). For dairy farms that milked <200 cows, the presence of swine on the farm was associated with 5.8 times greater odds of isolation of MR NASM in BTM (*P* = 0.05), but this association was not identified for larger herds (Table 3). There is little information about dairy farms and associations of other types of livestock (e.g., swine) carrying MR NASM (Schauer et al., 2021). Some information was provided about livestock MRSA (i.e., largely about multilocus ST398) that demonstrated an association with pigs, veal calves, or cows (Feingold et al., 2012). Pigs can serve as reservoirs of non-*mecA*-mediated oxacillin-resistant *S. aureus* phenotypes causing hard-to-treat diseases in humans that are referred to as borderline oxacillin-resistant *S. aureus* (**BORSA**). In this study we found only 4 non-*mecA*-mediated oxacillin-resistant *S. aureus*. Santos et al. (2021) described that the occurrence of BORSA phenotypes is probably underestimated in livestock when considering *mecA* as the main target to screen methicillin-resistant staphylococci, and it was the reason why we did not screen only using *mecA*. Other researchers have suggested that there is a notable risk factor for nasal carriage of livestock MRSA for persons with and without direct contact with livestock, such as direct contact with pigs and cows and living in a rural location, precluding valid inferences of absolute or relative risks (Locatelli et al., 2017; Schnitt and Tenhagen, 2020).

**Table 3:** Methicillin-resistant (MR) non-*aureus* staphylococci and mammaliicocci (NASM) positive test rates in bulk tank samples and association with presence of swine per herd size

Herd size category	Presence of swine^1^	MR NASM + N (%)	MR NASM − N (%)	Total	*P*-value^2^	Odds ratio (95% CI)
≤200 cows	Yes	2 (20.0%)	8 (80.0%)	10	0.049	5.8 (1.01–33.39)
	No	6 (4.1%)	139 (95.9%)	145		
		8 (5.2%)	147 (94.8%)	155		
						
>200 cows	Yes	0 (0.0%)	4 (100.0%)	4	0.513	2.8 (0.13–57.8)
	No	5 (3.5%)	136 (96.4%)	141		
		5 (3.4%)	140 (96.5%)	145		

1 Dairy farms that had swine as another livestock type.

2 Fisher's exact test.

Even considering that no MRSA was found in this study and only 4.6% of MRSA were found in US pig farms before (Smith et al., 2013), the global impact of MRSA on human and animal health continues (Becker, 2021). In fact, Becker (2021) stated that the impact of coagulase-negative staphylococci and coagulase-positive non-*S. aureus* complex species, as well as the role of macrococci as a source for methicillin resistance–encoding genetic elements in *S. aureus*, are only scantily investigated. Many aspects of the genetic basis, origin, distribution, and transmission of livestock-associated MR NASM are still poorly understood.

Schauer et al. (2021) emphasized that *M. sciuri* group are common in ruminants and they contain a large exchangeable antimicrobial gene reservoir for other closely related bacterial species (e.g., NASM or *S. aureus*), which may lend itself to bidirectional interspecies transmission. Future research should assess the new *M. sciuri* group in terms of its relationship to dairy farms and other types of livestock (e.g., swine) and the likelihood of exposure to methicillin resistance including isolates originating from different animal species as well as other countries to provide more details for this group of bacteria with a larger sample size.

Based on molecular results, we demonstrated a very low prevalence (<5%) of recovery of MR NASM from BTM samples collected from farms in the Upper Midwest. Despite years of use of β-lactams on farms, the prevalence of MR staphylococci appears to be reasonably stable in the United States. Future studies are encouraged to further explore MR NASM and their risk factors in dairy herds and other types of livestock.
